# Abdominal physical examinations in early stages benefit critically ill patients without primary gastrointestinal diseases: a retrospective cohort study

**DOI:** 10.3389/fmed.2024.1338061

**Published:** 2024-04-09

**Authors:** Xiao Cui, Yu Shi, Xinlei He, Mingyuan Zhang, Hua Zhang, Jianhong Yang, Yuxin Leng

**Affiliations:** ^1^Department of Intensive Care Units, Peking University Third Hospital, Beijing, China; ^2^School of Mechanical Engineering, University of Science and Technology Beijing, Beijing, China; ^3^Department of Research Center of Clinical Epidemiology, Peking University Third Hospital, Beijing, China

**Keywords:** abdominal physical examination, mortality, machine learning, predictive model, intensive care units

## Abstract

**Background:**

Gastrointestinal (GI) function is critical for patients in intensive care units (ICUs). Whether and how much critically ill patients without GI primary diseases benefit from abdominal physical examinations remains unknown. No evidence from big data supports its possible additive value in outcome prediction.

**Methods:**

We performed a big data analysis to confirm the value of abdominal physical examinations in ICU patients without GI primary diseases. Patients were selected from the Medical Information Mart for Intensive Care (MIMIC)-IV database and classified into two groups depending on whether they received abdominal palpation and auscultation. The primary outcome was the 28-day mortality. Statistical approaches included Cox regression, propensity score matching, and inverse probability of treatment weighting. Then, the abdominal physical examination group was randomly divided into the training and testing cohorts in an 8:2 ratio. And patients with GI primary diseases were selected as the validation group. Several machine learning algorithms, including Random Forest, Gradient Boosting Decision Tree, Adaboost, Extra Trees, Bagging, and Multi-Layer Perceptron, were used to develop in-hospital mortality predictive models.

**Results:**

Abdominal physical examinations were performed in 868 (2.63%) of 33,007 patients without primary GI diseases. A significant benefit in terms of 28-day mortality was observed among the abdominal physical examination group (HR 0.75, 95% CI 0.56–0.99; *p* = 0.043), and a higher examination frequency was associated with improved outcomes (HR 0.62, 95%CI 0.40–0.98; *p* = 0.042). Machine learning studies further revealed that abdominal physical examinations were valuable in predicting in-hospital mortality. Considering both model performance and storage space, the Multi-Layer Perceptron model performed the best in predicting mortality (AUC = 0.9548 in the testing set and AUC = 0.9833 in the validation set).

**Conclusion:**

Conducting abdominal physical examinations improves outcomes in critically ill patients without GI primary diseases. The results can be used to predict in-hospital mortality using machine learning algorithms.

## Graphical Abstract

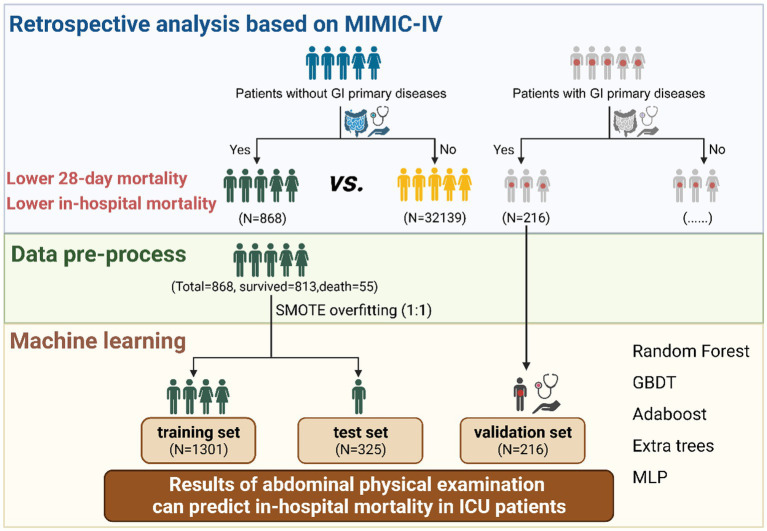

## Introduction

Gastrointestinal (GI) problems are common in intensive care units (ICUs) and are usually associated with poor outcomes in critically care patients (1–3). The GI tract acts as the “motor” of gut-derived sepsis and plays an important role in promoting the progression of multiple organ dysfunctions (MODS) (4–6). Nevertheless, GI dysfunction has not gained considerable attention compared with other organ dysfunctions. Widely used score systems describing patients’ conditions in intensive care units (ICUs) have not considered the GI system, such as Sequential Organ Failure Assessment (SOFA) scores and Acute Physiology and Chronic Health Evaluation (APACHE) scores (7, 8).

To emphasize GI dysfunction as a part of MODS and offer a better-scaled system, the Working Group on Abdominal Problems (WGAP) of the European Society of Intensive Care Medicine (ESICM) first proposed a grading system for GI dysfunction in critical illnesses named Acute Gastrointestinal Injury (AGI) (9). The introduction of standardized descriptions of AGI and GI symptoms has markedly promoted the development of related clinical research. The AGI grading system can effectively assess the severity of GI dysfunction and its grade is closely related to clinical outcomes (10–12). The sum of GI symptoms, including vomiting, diarrhea, and GI bleeding, etc., can independently predict all-cause mortality in ICU patients (10, 13). These findings provide clinical evidence for the importance of performing AGI evaluations in the ICU.

For mechanically ventilated and/or sedated patients, it is more difficult to obtain feedback regarding abdominal discomfort. Conducting objective parameter monitoring and abdominal physical examinations are the two options for identifying AGI in the early stages. Intra-abdominal pressure (IAP) is a unique objective indicator for AGI assessment but is not compulsory for all patients in the ICU (14). Intra-abdominal hypertension (IAH) is correlated with poor outcomes, but mild and transient elevation of IAP is “permissible” in certain cases (15). Because of the different perspectives, IAP is mainly measured in patients with GI primary pathology, such as those undergoing abdominal surgery and with GI primary diseases, and relevant data are limited (9, 16). However, evidence has shown that the outcome of patients with secondary AGI is worse than that of patients with primary GI disease (17). This phenomenon indicates that early assessment of GI function should be performed in all patients in the ICU, especially in those without obvious abnormalities at admission.

Abdominal physical examination provides quick information regarding the GI tract and offers assistance for further clinical management in the ICU. Compared with IAP measurements, abdominal physical examinations are simple, economical, and more easily performed by clinicians and nurses. For mechanically ventilated and/or sedated patients, abdominal physical examinations can provide information on GI function and are feasible. A study revealed that clinicians paid the most attention to complaints of bowel distension and bowel sounds in conscious and unconscious patients, respectively (18). Alteration of bowel sounds as a classic abnormal GI sign was reported to be significantly associated with mortality (19). Taken together, the abdominal physical examinations may benefit from following the progression of illness at the bedside, but there is no conclusive evidence to prove its benefit on ICU patient outcomes.

As abdominal physical examinations can reflect illness progression, it is worthy to consider abdominal physical examinations results as a predictor of the model. To date, there’s no literature supporting its additive value in outcome prediction. The logistic regression algorithm was the traditional model used in previous clinical research, but underfitting was performed because of the limited sample size and limited variables. In recent decades, machine learning methods, such as Random Forest, Gradient Boosting Decision Tree, Adaboost, Extra Trees, Bagging, and Multilayer Perceptron algorithms, have been developed and applied successfully in many types of areas (20–24). Machine learning can easily incorporate a large number of variables and improve model accuracy through feature selection, data preprocessing, etc. and has great potential in clinical research and practice.

Therefore, to identify the beneficial effect of abdominal physical examinations for all ICU patients, we first designed a retrospective study of ICU patients without primary GI diseases to clarify the benefit of abdominal physical examinations in patients without GI diseases. Second, to further study its possible additive value for mortality prediction, we tried to develop a prediction model using machine learning in patients without GI diseases, which could be extended to all patients with or without indications for early GI monitoring in the ICU.

## Methods

### Data source

The data involved in this study were obtained from a large publicly available dataset called the Medical Information Mart for Intensive Care (MIMIC) -IV database, which was developed by the Laboratory for Computational Physiology at MIT. MIMIC-IV (Version 1.0) contains comprehensive data on patients admitted to the critical care units of the Beth Israel Deaconess Medical Center between 2008 and 2019 (25). The study was conducted according to the Reporting of Studies Conducted using Observational Routinely Collected Health Data (RECORD) statement, and was reported in line with the STROCSS criteria (26, 27). One of our authors obtained access to and was responsible for data extraction (certification number 53051604).

### Participants

All the patients in MIMIC-IV aged ≥18 years without primary GI diseases (without direct insult to the GI tract and previous history of GI diseases) were enrolled for retrospective analysis and the development of predictive models (9). Among the patients, those who underwent abdominal palpation and auscultation within 48 h after ICU admission were allocated to the abdominal physical examination group, whereas the others were allocated to the no abdominal physical examination group. Adult patients with primary GI diseases who underwent physical examination were also included in the validation of the predictive models. Primary GI diseases were identified according to the International Classification of Diseases 9th Edition (ICD-9) code and the International Classification of Diseases 10th Edition (ICD-10) codes from MIMIC-IV. Patients who spent less than 48 h in the ICU or had missing outcome values were excluded. For those who had multiple admissions to the ICU, only the data from the first ICU admission were included in the analysis.

### Variable extraction

Baseline characteristics and abdominal physical examinations within 48 h of ICU admission were extracted using a structured query language (SQL), as shown in Table 1; Supplementary Table S1. Baseline characteristics included age, sex, weight, ICU type, Sequential Organ Failure Assessment (SOFA) score, and Simplified Acute Physiology Score II (SAPS II) score. The SOFA score was calculated as the sum of the maximum values for each sub-score within 24 h of admission. Not only vital signs, including the mean arterial pressure (MAP), heart rate, temperature (°C), and respiratory rate, but also laboratory variables, including white blood cell (WBC) count, hemoglobin, platelet counts, and the content of various elements, including sodium, potassium, chloride, creatinine, and urea nitrogen, which were measured during the first 24 h in the ICU, were selected from the dataset. Moreover, the Charlson comorbidity index and comorbidities including congestive heart failure, cerebrovascular disease, chronic pulmonary disease, liver-related comorbidity, kidney-related comorbidity, and malignancy were extracted. In addition, we extracted the counts of abdominal physical examinations during ICU stay and calculated the average frequency of abdominal physical examinations (ratio of abdominal physical examination counts to the length of ICU stay).

**Table 1:** Baseline characteristics.

Variables	Original cohort			
	Abdominal physical examination	No abdominal physical examination	*p*	SMD
*N*	868	32,139		
Age	57.27 (17.92)	65.35 (16.81)	<0.001	0.465
Gender, male (%)	545 (62.80)	18,307 (56.96)	<0.001	0.119
Weight (kg)	81.59 (20.85)	81.68 (25.00)	0.918	0.004
Types of ICU (%)			<0.001	0.585
SICU	212 (24.42)	4,713 (14.66)		
CVICU	209 (24.08)	7,824 (24.34)		
TSICU	200 (23.04)	3,911 (12.17)		
MICU	129 (14.86)	4,850 (15.10)		
MICU/SICU	67 (7.72)	4,303 (13.39)		
CCU	29 (3.34)	3,912 (12.17)		
NICU	22 (2.53)	2,626 (8.17)		
*Severity of illness*				
SOFA score	3.41 (2.20)	3.56 (2.43)	0.074	0.064
SAPS II score	32.31 (13.43)	35.35 (13.39)	<0.001	0.227
Charlson comorbidity index	4.17 (2.96)	5.47 (2.93)	<0.001	0.439
*Comorbidities, n (%)*				
Congestive heart failure	129 (14.86)	8,535 (26.56)	<0.001	0.292
Chronic pulmonary disease	172 (19.81)	7,560 (23.52)	0.012	0.090
Cerebrovascular disease	180 (20.74)	5,976 (18.59)	0.012	0.054
Renal disease	92 (10.60)	5,949 (18.51)	<0.001	0.226
Mild liver disease	72 (8.29)	1,834 (5.71)	0.002	0.102
Severe liver disease	21 (2.42)	870 (2.71)	<0.001	0.018
Malignant cancer	62 (7.14)	3,742 (11.64)	<0.001	0.155
*Vital signs*				
Heart rate (bpm)	84.99 (15.50)	83.81 (15.29)	0.025	0.077
MAP (mmHg)	79.78 (10.28)	78.68 (10.48)	0.002	0.106
Respiratory rate (bpm)	18.92 (3.56)	18.99 (3.63)	0.542	0.021
Temperature (°C)	36.98 (0.47)	36.86 (0.50)	<0.001	0.264
*Laboratory tests*				
WBC (×10^9^/L)	12.75 (11.55)	12.46 (9.00)	0.352	0.028
Hemoglobin (×10^12^/L)	11.35 (2.07)	11.00 (2.08)	<0.001	0.172
Platelets (×10^9^/L)	207.77 (91.82)	203.48 (95.95)	0.194	0.046
Sodium (mmol/L)	138.03 (3.82)	138.38 (4.61)	0.028	0.082
Potassium (mmol/L)	4.13 (0.57)	4.26 (0.59)	<0.001	0.211
Creatinine (mg/dL)	1.17 (1.41)	1.32 (1.41)	0.003	0.104
BUN (mg/dL)	19.25 (16.30)	23.64 (19.13)	<0.001	0.247

SICU, surgical intensive care unit; CVICU, cardiac vascular intensive care unit; TSICU, trauma surgical intensive care unit; MICU, medical intensive care unit; MICU/SICU, medical/surgical intensive care unit; CCU, coronary care unit; NICU, neuro surgical intensive care unit; SOFA, sequential organ failure assessment; SAPS II, simplified acute physiology score II; MAP, mean arterial pressure; WBC, white blood cell; BUN, blood urea nitrogen.

### Primary and secondary outcomes

The primary outcome was the 28-day mortality. Secondary outcomes included 60-day and 90-day in-hospital mortality and length of ICU stay (LOS).

### Statistical analysis

Continuous variables are presented as means (standard deviations) or medians [interquartile ranges (IQRs)], and categorical variables were presented as total numbers and percentages. Comparisons between groups were made using the Chi-squared test for categorical variables and the t-test or Mann–Whitney *U* test for continuous variables, as appropriate.

The Cox proportional hazards model was used to characterize the relationship between abdominal physical examinations and outcomes. To determine the potential covariates, we first performed a univariate Cox analysis of the baseline data (Table 1). The parameters correlated with 28-day mortality (*p* < 0.10) and clinically judged significant by experts were finally included in the multi-Cox analysis. The covariables were age, gender, ICU admission types of ICU, baseline SAPS II score, SOFA score, Charlson comorbidity index, comorbidities, vital signs (heart rate, mean arterial pressure, respiratory rate, and temperature), and initial laboratory tests (hemoglobin, sodium, potassium, creatinine, and blood urea nitrogen). In addition, given that the effect of GI physical examinations may vary according to the inspection frequency during hospitalization, we also performed an additional analysis to show the association between the average frequency of abdominal physical examinations and mortality outcome.

We conducted propensity score matching (PSM) and inverse probability of treatment weighting (IPTW) analysis to adjust the covariates to reduce the influence of data biases and confounding variables to obtain more reasonable comparisons between the experimental and control groups (28, 29). Thus, a 1:1 nearest neighbor matching with a caliper width of 0.05 was applied in our study. Standardized mean differences (SMDs) and *p*-values were calculated to evaluate the effectiveness of PSM and IPTW (30). The baseline characteristics and SMDs of the two groups after PSM and IPTW were shown in Supplementary Tables S2, S3. Moreover, for the matched cohort, we analyzed the long-term prognosis by plotting survival curves and testing the significance of mortality in the sample by Log Rank (Mantel–Cox) methods. After PSM and IPTW, we performed the Cox regression for further analysis.

All statistical analyses were performed with the Jupyter Notebook (Anaconda 3) and RStudio (version 4.2.0). A *p*-value was taken as statistically significant at *p* < 0.05 (two-sided).

### Machine learning methods

To provide specific evidence on the value of abdominal physical examinations in predicting in-hospital mortality in critically ill patients, we performed machine learning studies. The above-mentioned baseline variables, together with the results of abdominal palpation and auscultation were included as predictors. Specifically, the baseline variables included age, gender, ICU admission types of ICU, baseline SAPS II score, SOFA score, Charlson comorbidity index, comorbidities, vital signs (heart rate, mean arterial pressure, respiratory rate, and temperature), and initial laboratory tests (hemoglobin, sodium, potassium, and blood urea nitrogen). Namely, according to whether the examinations were normal (Supplementary Table S1), as long as one of the four items had an abnormal result, it was defined as physically abnormal. Since the data set was imbalanced, where the ratio of surviving patients to deceased patients in the patient data of the abdominal physical examination group was approximately 15, the synthetic minority oversampling technique (SMOTE) algorithm (31), which is an oversampling method, was used to pre-process the data. To validate the model overfitting problem, the data of the validation group were left unprocessed to retain their imbalance characteristics, and the performance of the trained machine learning model on the imbalanced data set was observed and evaluated. The patient data of the abdominal physical examination group was randomly divided, of which 80% was used as the training set and 20% as the testing set. In addition, to verify the models’ accuracy, robustness, and generalizability, excluded patients with primary GI diseases were selected to test the models as a validation group. For the machine learning classification algorithms, Random Forest, Gradient Boosting Decision Tree (GBDT), Adaboost, Extra Trees, Bagging, and Multilayer Perceptron (MLP) were applied. During training, the model was first optimized for a single parameter. According to the change trend results of each parameter and model performance, the grid optimization search algorithm was used to optimize the multi-parameter overall model, and the final predictive model was obtained. Accuracy, precision, recall, F1-score, and area-under-curve (AUC) were selected to evaluate the performance of algorithms.

## Results

### Study population and the baseline characteristics

**Figure 1:**
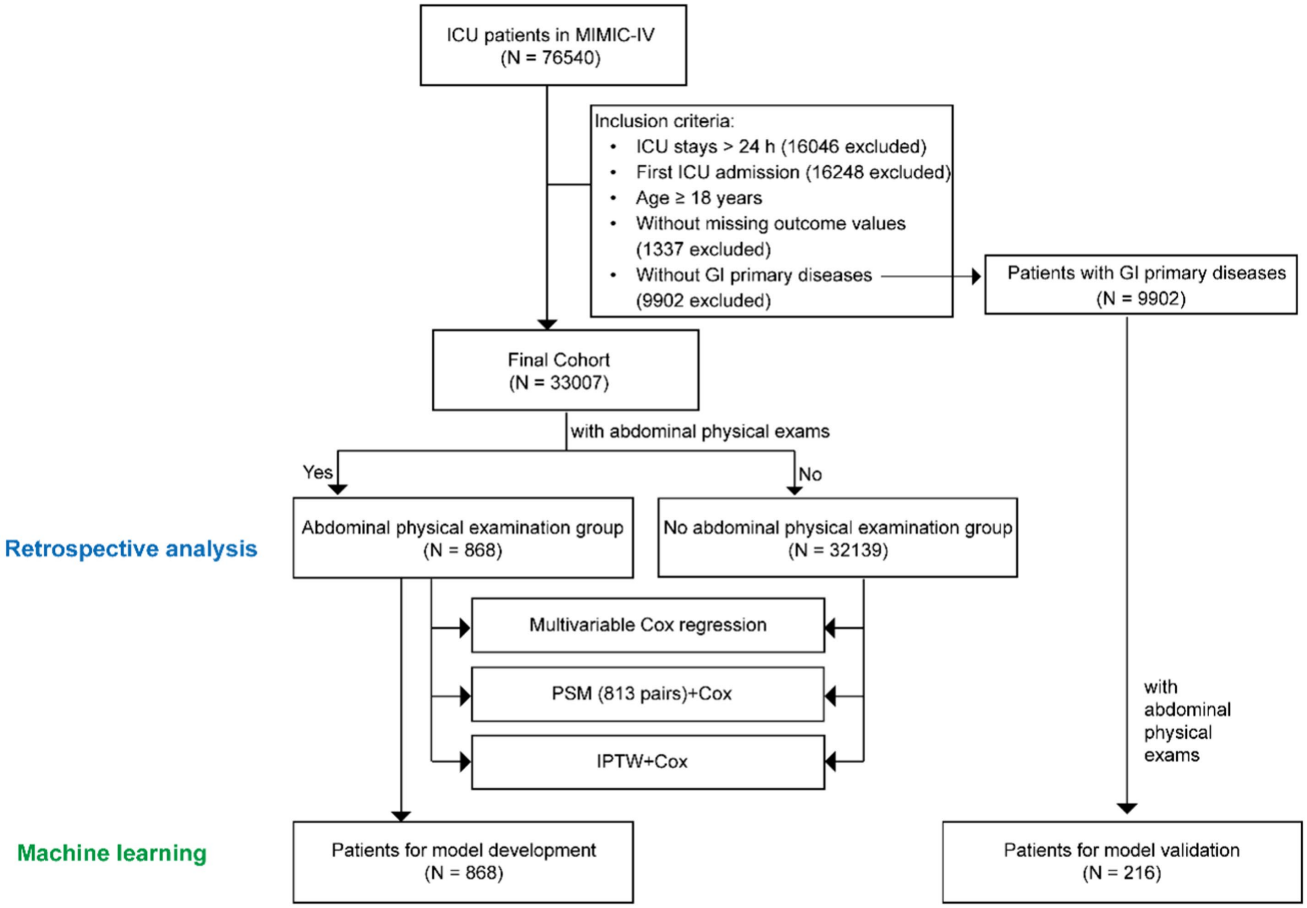
Study population. MIMIC, Medical Information Mart for Intensive Care.

According to the exclusion and inclusion criteria, 33,007 patients without primary GI diseases were enrolled. The study population was shown in Figure 1. Among them, 868 patients (2.63%) underwent bedside abdominal physical examinations in the first 48 h after ICU admission, while the remaining 32,139 patients did not. A retrospective analysis was performed for both groups. Multivariate Cox regression, PSM, IPTW were used to demonstrate the benefit of performing abdominal physical examinations for patients without indications by balancing the confounding factors. To further investigate the potential additive value of abdominal examinations in predicting mortality for all ICU patients, we tried to develop a prediction model using machine learning in patients without GI diseases. The abdominal physical examination group (*N* = 868) was used for model development, and 216 patients with GI primary diseases were used for model validation.

Table 1 showed the baseline characteristics between the groups with and without abdominal physical examination groups. Patients in the SICU (24.42% vs. 14.66%) and TSICU (23.04% vs. 12.17%) received more attention on GI function. The abdominal physical examination group had lower SAPS II scores on admission [32.31(±13.43) vs. 35.35(±13.39); *p* < 0.001] and had a lower Charlson comorbidity index [4.17 (2.96) vs. 5.47 (2.93)] and fewer comorbidities overall.

### Application of abdominal physical examinations improved the primary outcome

To clarify the association between the application of abdominal physical examinations and the 28-day mortality, we used the multivariate Cox proportional hazard model. The results demonstrated a significant beneficial effect of bedside abdominal physical examinations on 28-day mortality (Table 2), with a hazard ratio (HR) of 0.75 (95%CI 0.56–0.99; *p* = 0.043). The PSM analysis generated 813 pairs. The imbalance in covariates between the two groups was significantly reduced after PSM (Supplementary Table S2), and the association remained robust (Table 2). The application of abdominal physical examinations was associated with improved 28-day mortality after PSM (HR 0.62, 95% CI 0.62–0.92; *p* = 0.017) and IPTW (HR 0.65, 95% CI 0.43–0.98; *p* = 0.042).

**Table 2:** Primary and secondary outcomes analysis with three different statistical methods.

Outcomes	HR	CI of HR		*p*
		2.5%	97.5%	
*Cox regression* ^*^				
28-day mortality	0.75	0.56	0.99	0.043
60-day mortality	0.74	0.57	0.97	0.032
90-day mortality	0.74	0.56	0.96	0.025
In-hospital mortality	0.75	0.56	0.99	0.034
*PSM + Cox regression*				
28-day mortality	0.62	0.42	0.92	0.017
60-day mortality	0.59	0.40	0.85	0.005
90-day mortality	0.59	0.41	0.85	0.005
In-hospital mortality	0.62	0.42	0.92	0.017
*IPTW + Cox regression*				
28-day mortality	0.65	0.43	0.98	0.042
60-day mortality	0.65	0.43	0.98	0.042
90-day mortality	0.65	0.44	0.96	0.031
In-hospital mortality	0.65	0.43	0.98	0.005

^*^Covariables included age, gender, ICU admission types of ICU, baseline SAPS II score, SOFA score, Charlson comorbidity index, vital signs (heart rate, mean arterial pressure, respiratory rate, and temperature), and initial laboratory tests (hemoglobin, sodium, potassium, creatinine, and blood urea nitrogen). PSM, propensity score matching; IPTW, inverse probability of treatment weight; HR, hazard ratio; CI: confidence interval.

We also conducted an additional study to evaluate the association between the average frequency of abdominal physical examinations and outcomes (Table 3). In the abdominal physical examination group, the average frequency of abdominal physical examinations was 0.89 counts/day (IQR 0.49–1.61 counts/day). And the multivariate Cox model showed a significant beneficial effect of more abdominal physical examinations on 28-day mortality (HR 0.62, 95% CI 0.40–0.98; *p* = 0.042).

**Table 3:** Association of average frequency of abdominal physical examinations during ICU and outcomes.

Outcomes	HR	CI of HR		*p*
		2.5%	97.5%	
*Primary outcomes*				
28-day mortality	0.62	0.40	0.98	0.042
*Secondary outcomes*				
60-day mortality	0.61	0.39	0.94	0.025
90-day mortality	0.60	0.39	0.92	0.020
In-hospital mortality	0.63	0.41	0.99	0.044

Average frequency of abdominal physical examinations = counts of abdominal physical examinations during ICU stays/length of ICU stays. Covariables included age, gender, ICU admission types of ICU, baseline SAPS II score, SOFA score, Charlson comorbidity index, vital signs (heart rate, mean arterial pressure, respiratory rate, and temperature), and initial laboratory tests (hemoglobin, sodium, potassium, creatinine, and blood urea nitrogen).

Analyses showed a beneficial effect of the application of bedside abdominal physical examination in terms of 28-day mortality, and the higher average frequency of examinations was associated with lower 28-day mortality.

### Application of abdominal physical examinations to improved secondary outcomes

**Figure 2:**
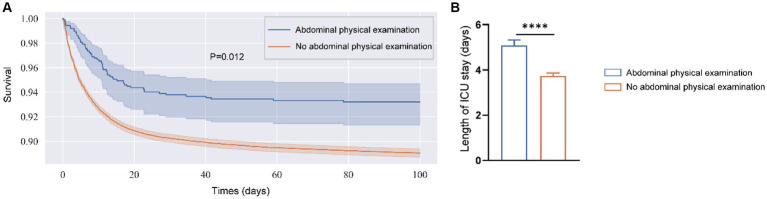
Kaplan–Meier survival curve **(A)** and length of stay in ICU **(B)** for two groups after PSM. **(A)** Shaded regions are 95% confidence intervals. **(B)** Was determined by unpaired *t*-test and data were shown in mean + SEM. ^****^p < 0.001.

The application of abdominal physical examinations was also investigated for 60-day, 90-day, and in-hospital mortality (Table 2). The results showed that performing bedside abdominal physical examinations associated with improved 60-day mortality (HR 0.74, 95% CI 0.57–0.97; *p* = 0.032), 90-day mortality (HR 0.74, 95% CI 0.57–0.96; *p* = 0.025), and in-hospital mortality (HR 0.75, 95% CI 0.56–0.99; *p* = 0.034). The PSM model and IPTW model led to the same conclusion. Furthermore, the more average frequency was associated with lower 60-day (HR 0.61, 95% CI 0.39–0.94; *p* = 0.025), 90-day (HR 0.60, 95% CI 0.39–0.92; *p* = 0.020), and in-hospital mortality (HR 0.63, 95% CI 0.41–0.99; *p* = 0.44) (Table 3). After PSM, the Kaplan–Meier (KM) survival curve indicated the abdominal physical examinations had a beneficial effect on the increased survival time (Figure 2). In addition, although the abdominal physical examination group had lower severity scores (Table 1), patients had longer lengths of stay in the ICU (Figure 2), which might be attributed to more care/treatment from clinicians.

### The alterations of the following interventions based on the abdominal physical examinations results

**Figure 3:**
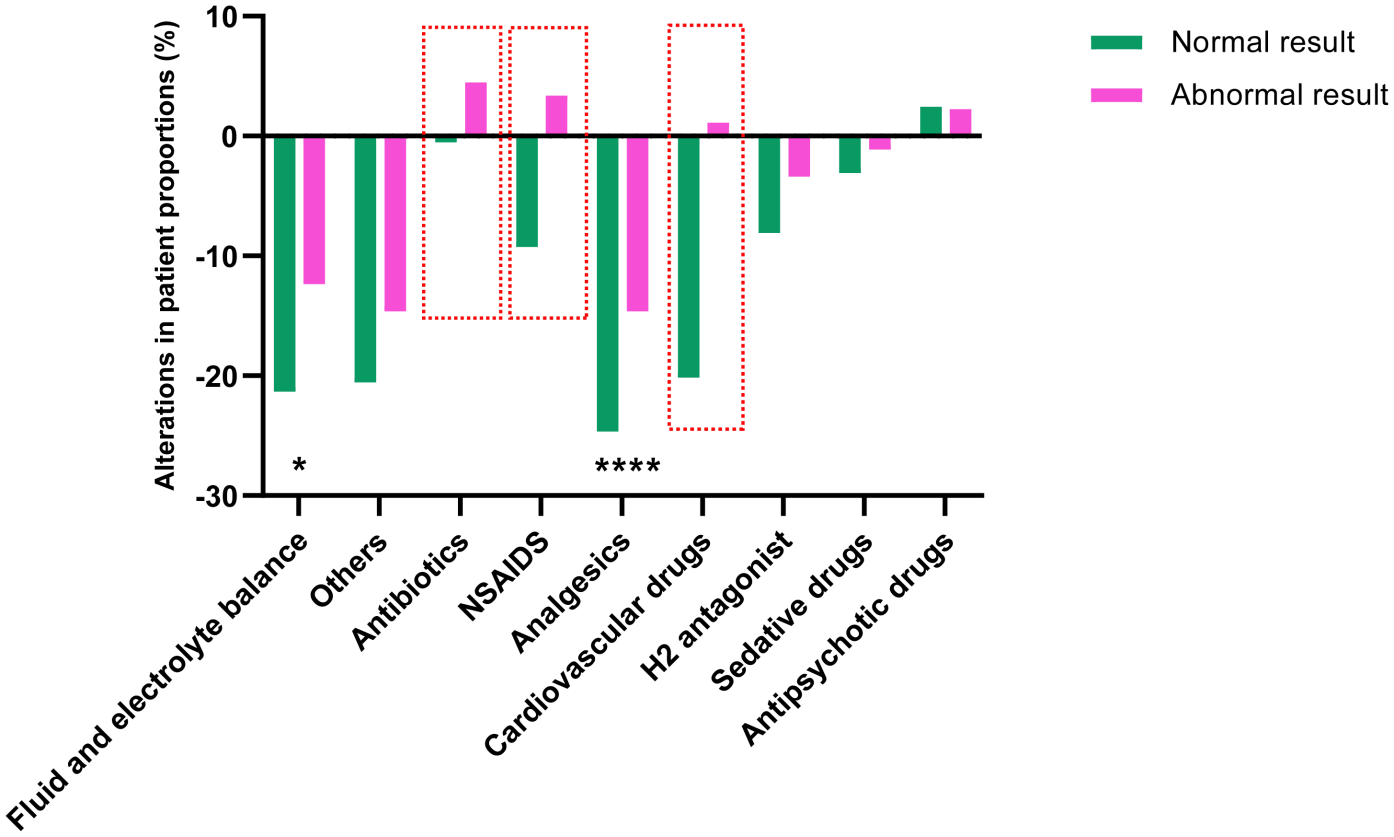
The alteration of following interventions after abdominal physical examinations. The height and the direction of the bars represent the differences of the proportion of patients taking representative drugs. Comparisons between groups were made using the chi-squared test. The red boxes indicate the drug with the most significant differences between the two groups. ^*^p < 0.05 and ^****^p < 0.001.

To investigate how abdominal physical examinations affected subsequent therapeutic strategies, we compared the alteration of interventions after performing examinations (within 48 h), which might be directly related with the results of examination. We found that among the 868 detected patients, the examination results of 89 patients were abnormal, and the examination results of 779 patients were normal. Patients (%) with antibiotics, NSAIDs, and cardiovascular drugs were increased in the Abnormal result group, while decreased in the Normal result group after examination. This could be due to the abnormal results indicating the occurrence of abdominal infection or GI dysfunction. Clinicians attempt to control abdominal infection, relief pain and improve GI blood perfusion by antibiotics, NSAIDs and cardiovascular drugs. In addition, although all the patients (%) receiving fluid and electrolyte management and analgesics decreased, the proportion of patients in the Normal result group decreased significantly compared to that in the Abnormal result group. This suggests that when the results indicate abnormality, the clinicians should pay more attention on the fluids and electrolytes management and pain management (see Figure 3).

### Development and validation of in-hospital mortality predictive models based on the results of abdominal physical examinations

Data from the abdominal physical examination group was used to develop in-hospital mortality prediction models. The data was highly imbalanced in terms of mortality (survived = 813, death = 55). To achieve better prediction accuracy on such a data set, we used SMOTE overfitting (1:1) to pre-process the patient data in the abdominal physical examination group, while leaving the validation set data unprocessed. Ultimately, data from a total of 1,626 patients without GI primary diseases (survived = 813) were randomly divided into the training set for modeling and the testing set for validation in the ratio of 8:2. Then, the performance of the model was evaluated with the validation set data (*n* = 216, survived = 181). The final results of the testing set and the validation set attained with these models (Random Forest, XGBoost, Adaboost, Extra Trees, Bagging, MLP) an illustrated in Table 4. Supplementary Figures S1, S2 showed the receiver operating characteristic (ROC) curves of the models in the testing cohort and the validation cohort. The random forest model and the MLP model performed well in predicting in-hospital mortality, with AUC values of 0.9514 and 0.9548 in the testing set, and 0.9901 and 0.9833 in the validation set, respectively. But the MLP model had less storage space than the random forest, with only 151 kB (Supplementary Table S4). Taking model performance and storage space, the MLP model performed best.

**Table 4:** Evaluation of machine learning algorithms.

Model	Test set					Validation set				
	Accuracy	Precision	Recall	F1-Score	AUC	Accuracy	Precision	Recall	F1-Score	AUC
Random Forest	0.95	0.99 (0) 0.90 (1)	0.91 (0) 0.99 (1)	0.95 (0) 0.94 (1)	0.9514	0.95	1.00 (0) 0.90 (1)	0.91 (0) 1.00 (1)	0.95 (0) 0.95 (1)	0.9901
GBDT	0.92	0.97 (0) 0.87 (1)	0.88 (0) 0.97 (1)	0.93 (0) 0.92 (1)	0.9270	0.95	0.99 (0) 0.91 (1)	0.92 (0) 0.99 (1)	0.96 (0) 0.95 (1)	0.9913
Adaboost	0.93	0.98 (0) 0.87 (1)	0.88 (0) 0.98 (1)	0.93 (0) 0.92 (1)	0.9304	0.93	0.98 (0) 0.87 (1)	0.88 (0) 0.98 (1)	0.93 (0) 0.92 (1)	0.9079
Extra Trees	0.92	0.96 (0) 0.88 (1)	0.89 (0) 0.96 (1)	0.93 (0) 0.92 (1)	0.9258	0.92	0.96 (0) 0.88 (1)	0.89 (0) 0.96 (1)	0.93 (0) 0.92 (1)	0.9517
Bagging	0.92	0.99 (0) 0.85 (1)	0.86 (0) 0.99 (1)	0.92 (0) 0.91 (1)	0.9225	0.92	0.99 (0) 0.85 (1)	0.86 (0) 0.99 (1)	0.92 (0) 0.91 (1)	0.9752
MLP	0.95	1.00 (0) 0.90 (1)	0.91 (0) 1.00 (1)	0.95 (0) 0.95 (1)	0.9548	0.95	1.00 (0) 0.90 (1)	0.91 (0) 1.00 (1)	0.95 (0) 0.95 (1)	0.9833

^*^GBDT, gradient boosting decision tree; MLP, multilayer perceptron.

## Discussion

In our study, we demonstrated that the application of bedside abdominal physical examinations in patients without original GI causes was associated with significantly lower 28-day, 60-day, 90-day and in-hospital mortality. And this beneficial effect was associated with a higher frequency of examination. After the adjustment of confounding factors, the results were found to be robust. Therefore, we used machine learning methods to develop a model for the abdominal physical examination group without GI primary diseases to predict mortality and validated the robustness and extensibility of the model in patients with GI primary disease. Analysis results show that performing abdominal physical examinations within 48 h is valuable in improving patient outcomes, especially for those without GI primary diseases. Machine learning using physically examined results can be used to predict in-hospital mortality in critically ill patients.

Gut protection in ICUs has recently gained considerable attention. Whether GI evaluation should be added to the scoring system, such as the SOFA, has been widely discussed (13, 19). In 2013, Reintam Blaser et al. (19) showed that the appearance of GI symptoms during the first week in the ICU was associated with poor outcomes in patients requiring mechanical ventilation, among which absent bowel sounds and GI bleeding showed the most significant association with the 28-day mortality. But due to missing data and unclear definitions, they could not develop a more accurate scoring system with GI symptoms on the admission day compared with the SOFA score (AUROC: 0.706 vs. 0.703). In 2019, Padar et al. (13) conducted a retrospective study to describe the incidence and outcome of GI failure and tried to evaluate the feasibility of adding GI-variable to the SOFA score. They found that approximately 10% of ICU patients (413/3,959) had GI failure on the first day, which was accompanied by longer ICU stays and higher mortality. The number of GI symptoms on ICU admission can independently predict mortality, similar to other SOFA sub-scores. When combined with the SOFA score, a higher number of GI symptoms increased the accuracy of the former as a predictor. These findings reveal that GI evaluation is important for patients in the ICU. The present study further strengthens the credibility of the evidence through big data analysis and machine learning-based modeling.

To objectively evaluate GI function is difficult in ICU, although the concept of AGI has been defined (16). As Deane et al. (32) discussed in their review, classic bedside examinations of bowel sounds and abdominal distension were important for initiating enteral nutrition in critically ill patients. In our study, we further extended their findings and found that early assessment of GI function through abdominal palpation and auscultation is helpful in achieving excellent performance in mortality prediction.

It should be noted that, new non-invasive, objective, sensitive, and explainable technologies can be anticipated. Although abdominal physical examinations were considered in the proposed predictive models, more objective, reproducible, non-invasive, and sensitive examinations for GI function are needed in the ICU. The assessment of GI symptoms and signs can be made more precise with artificial intelligence. For example, bowel sounds were considered in our study, but analysis of bowel sounds is subjective. Combined with acoustic signal processing techniques and machine learning methods, bowel sound detection and analysis will be more automatic and objective (33, 34). We developed equipment that can detect bowel sounds and intra-abdominal pressure in a sensitive, real-time, and non-invasive manner (35). It is believed that more intelligent, real-time, and non-invasive methods have promising demands for GI function examinations in the ICU. On the other hand, the underlying pathophysiology of GI failure is complicated, and the relevant monitoring technologies are limited (36, 37). Magnetoenterography is a non-invasive technique that detects gastrointestinal magnetic signals. It has high sensitivity, and a high signal-to-noise ratio compared with electrogastrography and electrointestinography, and has been used for mesenteric ischemia and damage to the intestinal microstructure (38–40).

Several limitations in this study should be noted. First, the examinations may affect the subsequent interventions and further influence the outcome. We did not make a robust causal inference between abdominal physical exams and prognosis. Advanced statistical approaches like marginal structural models may be useful for revealing causal relationships (41). Future studies need to take this into account. Second, our research is an observational study. More relevant and persuasive clinical trials are the gold standard for causal inference and required to confirm our findings and conclusions. Third, our study is a retrospective cohort study based on electronic health records (EHRs). Manually error records are unavoidable. Fourth, the items about abdominal physical examinations chosen from the MIMIC database were not based on objective observation, and the involved symptoms and signs are subjective. Fifth, given that application of abdominal physical examinations can improve outcomes, future studies are still needed to prove whether IAP measurement is necessary to become routine. Besides, our results were merely based on the MIMIC-IV database, and we did not perform external validation of our predictive model. It should be pointed out that if an external validation is carried out, the results will be more solid. In the future, it is necessary to conduct multi-center clinical studies to demonstrate the reliability of our conclusions and predictive models.

Despite these limitations, it can be observed that performing abdominal physical examinations can improve outcomes and results can predict mortality as part of predictors in ICU patients. The use of physical exams is better than abandoning routine assessment for GI function. Since the assessment of GI function remains indispensable to evaluating patients’ outcomes, future assessment score systems should include the GI system.

In conclusion, big data analysis on the MIMIC-IV database shows that patients without GI primary diseases could benefit from abdominal physical examinations, which highlights the essential role of the GI system in MODS. The predictive model with machine learning algorithms based on the results of abdominal physical examinations can effectively predict the mortality and be extended to all ICU patients, which has important and practical meaning in ICU. More objective, non-invasive, and sensitive tools for GI standardized assessment are expected to be developed.

## Data availability statement

The original contributions presented in the study are included in the article/Supplementary material, further inquiries can be directed to the corresponding authors.

## Ethics statement

The studies involving humans were approved by the Massachusetts Institute of Technology (Cambridge, MA) and Beth Israel Deaconess Medical Center (Boston, MA). The studies were conducted in accordance with the local legislation and institutional requirements. Written informed consent for participation was not required from the participants or the participants’ legal guardians/next of kin in accordance with the national legislation and institutional requirements.

## Author contributions

XC: Conceptualization, Formal analysis, Investigation, Visualization, Writing – original draft. YS: Data curation, Investigation, Methodology, Software, Validation, Visualization, Writing – original draft. XH: Data curation, Visualization, Writing – original draft. MZ: Data curation, Methodology, Resources, Software, Writing – original draft. HZ: Methodology, Writing – review & editing. JY: Conceptualization, Methodology, Resources, Supervision, Writing – review & editing. YL: Conceptualization, Funding acquisition, Resources, Supervision, Writing – review & editing.

## Glossary

GI: Gastrointestinal
MODS: Multiple organ dysfunctions
ICU: Intensive care units
SOFA: Sequential Organ Failure Assessment
APACHE: Acute Physiology and Chronic Health Evaluation
WGAP: Working Group on Abdominal Problems
ESICM: European Society of Intensive Care Medicine
AGI: Acute gastrointestinal injury
MIMIC: Medical Information Mart for Intensive Care
ICD-9: International Classification of Diseases 9th Edition
ICD-10: International Classification of Diseases 10th Edition
SQL: Structured query language
SAPS II: The Simplified Acute Physiology Score II
MAP: Mean arterial pressure
WBC: White blood cells
LOS: Length of ICU stay
IQRs: Interquartile ranges
PSM: Propensity score matching
IPTW: Inverse probability of treatment weighting
SMDs: Standardized mean differences
GBDT: Gradient Boosting Decision Tree
MLP: Multilayer Perceptron
ROC: Receiver operating characteristic curve
AUC: Area-under-curve
SICU: Surgical intensive care unit
CVICU: Cardiac vascular intensive care unit
TSICU: Trauma surgical intensive care unit
MICU: Medical intensive care unit
MICU/SICU: Medical/surgical intensive care unit
CCU: Coronary care unit
NICU: Neuro surgical intensive care unit
BUN: Blood urea nitrogen
HR: Hazard ratio
CI: Confidence interval
PPI: Proton pump inhibitors
NSAIDS: Nonsteroidal anti-inflammatory drugs
CRRT: Continuous renal replacement therapy
