# Supplementary material for: Abdominal physical examinations in early stages benefit critically ill patients without primary gastrointestinal diseases: a retrospective cohort study
Source: Front Med (Lausanne). 2024 Apr 9;11:1338061. doi: 10.3389/fmed.2024.1338061 (PMC11037245; doi:10.3389/fmed.2024.1338061)
Supplement: Supplementary file 1 [file Data_Sheet_1.docx]

**Supplementary materials**

**Supplementary tables**

**Table S1. The items of abdominal physical exams in MIMIC-IV**

| Itemid | Reports | Results |
| --- | --- | --- |
| 229594 | Bowel sounds present | Normal |
|  | Distended | Abnormal |
|  | Soft, nontender | Normal |
| 224673 | No abdominal complaints, normal bowel sounds | Normal |
|  | Reports crampy abdominal pain, diarrheal movements, active bowel sounds | Abnormal |
|  | Reports waves of crampy abdominal pain, active bowel sounds | Abnormal |
| 229453 | Adequate urine output | Normal |
|  | Bowel sounds present | Normal |
|  | Distended | Abnormal |
|  | Soft, nontender | Normal |
| 228698 | No abdominal complaints, normal bowel sounds | Normal |

**Table S2. Comparisons of baseline characteristics after propensity score matching**

| Variables | Matched cohort (After PSM) | | | |
| --- | --- | --- | --- | --- |
|  | Abdominal physical examination | No abdominal physical examination | p | SMD |
| N | 813 | 813 |  |  |
| age | 56.90 (18.05) | 57.77 (17.99) | 0.327 | 0.049 |
| Gender, male (%) | 514 (63.2) | 520 (64.0) | 0.797 | 0.015 |
| Weight(kg) | 81.63 (20.92) | 84.21 (24.79) | 0.023 | 0.113 |
| Types of ICU (%) |  |  | 0.672 | 0.119 |
| SICU | 204 (25.1) | 213 (26.2) |  |  |
| CVICU | 180 (22.1) | 187 (23.0) |  |  |
| TSICU | 193 (23.7) | 181 (22.3) |  |  |
| MICU | 122 (15.0) | 131 (16.1) |  |  |
| MICU/SICU | 67 (8.2) | 71 (8.7) |  |  |
| CCU | 27 (3.3) | 19 (2.3) |  |  |
| NICU | 20 (2.4) | 21 (1.1) |  |  |
| Severity of illness |  |  |  |  |
| SOFA score | 3.34 (2.15) | 3.34 (2.23) | 0.985 | 0.001 |
| SAPS II score | 31.95 (13.33) | 32.41 (13.43) | 0.486 | 0.035 |
| Charlson Comorbidity Index | 4.10 (2.98) | 4.14 (2.67) | 0.791 | 0.013 |
| Comorbidities, n (%) |  |  |  |  |
| Congestive heart failure | 109 (13.4) | 94 (11.6) | 0.294 | 0.056 |
| Chronic pulmonary disease | 159 (19.6) | 144 (17.7) | 0.373 | 0.047 |
| Cerebrovascular disease | 168 (20.7) | 166 (20.4) | 0.951 | 0.006 |
| Renal disease | 85 (10.5) | 77 (9.5) | 0.562 | 0.033 |
| Mild liver disease | 71 (8.7) | 71 (8.7) | 1 | <0.001 |
| Severe liver disease | 19 (2.3) | 23 (2.8) | 0.693 | 0.031 |
| Malignant cancer | 58 (7.1) | 53 (6.5) | 0.694 | 0.047 |
| Vital signs |  |  |  |  |
| Heart rate(bpm) | 85.02 (15.59) | 85.50 (15.53) | 0.535 | 0.031 |
| MAP (mmHg) | 80.02 (10.35) | 79.79 (10.78) | 0.666 | 0.021 |
| Respiratory rate (bpm) | 18.89 (3.54) | 19.07 (3.77) | 0.305 | 0.051 |
| Temperature (℃) | 36.99 (0.47) | 36.97 (0.48) | 0.583 | 0.027 |
| Laboratory tests |  |  |  |  |
| WBC (×10^9^/L) | 12.44 (5.62) | 12.84 (6.77) | 0.199 | 0.064 |
| Hemoglobin (×10^12^/L) | 11.42 (2.06) | 11.36 (2.02) | 0.619 | 0.025 |
| Platelets (×10^9^/L) | 209.59 (92.86) | 205.00 (92.94) | 0.319 | 0.049 |
| Sodium (mmol/L) | 138.02 (3.85) | 138.45 (4.21) | 0.030 | 0.108 |
| Potassium (mmol/L) | 4.13 (0.57) | 4.14 (0.51) | 0.717 | 0.018 |
| Creatinine (mg/dL) | 1.17 (1.44) | 1.20 (1.42) | 0.065 | 0.022 |
| BUN (mg/dL) | 19.05 (16.32) | 19.54 (15.00) | 0.529 | 0.031 |

*SICU Surgical Intensive Care Unit, CVICU Cardiac Vascular Intensive Care Unit, TSICU Trauma Surgical Intensive Care Unit, MICU Medical Intensive Care Unit, MICU/SICU Medical/Surgical Intensive Care Unit, CCU Coronary Care Unit, NICU Neuro Surgical Intensive Care Unit, SOFA Sequential Organ Failure Assessment, SAPS II Simplified Acute Physiology Score II, MAP mean arterial pressure, WBC white blood cell, BUN Blood Urea Nitrogen.

**Table S3. Comparisons of baseline characteristics after** **the inverse probability of treatment weighting**

| Variables | Matched cohort (After IPTW) | | | |
| --- | --- | --- | --- | --- |
|  | Abdominal physical examination | No abdominal physical examination | p | SMD |
| N | 32962.7 | 33027.1 |  |  |
| Age | 57.8 (17.4) | 65.4(16.8) | <0.001 | 0.443 |
| Gender, male (%) | 19033.9 (62.8) | 18815.8 (57.0) | 0.768 | 0.016 |
| Weight(kg) | 81.6 (20.7) | 81.7 (25.0) | 0.882 | 0.006 |
| Types of ICU (%) |  |  | <0.001 | 0.660 |
| SICU | 8268.3 (25.1) | 4843.0 (14.7) |  |  |
| CVICU | 7474.5 (22.7) | 8040.0 (24.3) |  |  |
| TSICU | 8738.9 (26.5) | 4018.0 (12.2) |  |  |
| MICU | 4511.0 (13.7) | 4985.9 (15.1) |  |  |
| MICU/SICU | 2367.7 (7.2) | 4418.5 (13.4) |  |  |
| CCU | 881.0 (2.7) | 4022.2 (12.2) |  |  |
| NICU | 721.5 (2.2) | 2699.5 (8.2) |  |  |
| Severity of illness |  |  |  |  |
| SOFA score | 3.4 (2.1) | 3.6 (2.4) | 0.081 | 0.073 |
| SAPS II score | 31.9 (13.4) | 35.4 (13.4) | <0.001 | 0.258 |
| Charlson Comorbidity Index | 4.3 (2.9) | 5.5 (2.9) | <0.001 | 0.412 |
| Comorbidities, n (%) |  |  |  |  |
| Congestive heart failure | 4889.2 (14.8) | 8772.9 (26.6) | <0.001 | 0.293 |
| Chronic pulmonary disease | 5893.1 (17.9) | 7771.7 (23.5) | 0.003 | 0.140 |
| Cerebrovascular disease | 7495.8 (22.7) | 6141.8 (18.6) | 0.078 | 0.102 |
| Renal disease | 3487.8 (10.6) | 6115.3 (18.5) | <0.001 | 0.226 |
| Mild liver disease | 2413.4 (7.3) | 1885.7 (5.7) | 0.079 | 0.065 |
| Severe liver disease | 627.7 (1.9) | 894.6 (2.7) | 0.152 | 0.054 |
| Malignant cancer | 2127.7 (6.5) | 3844.8 (11.6) | <0.001 | 0.182 |
| Vital signs |  |  |  |  |
| Heart rate(bpm) | 84.5 (15.5) | 83.8 (15.3) | 0.036 | 0.107 |
| MAP (mmHg) | 80.1 (10.2) | 78.7 (10.5) | 0.007 | 0.139 |
| Respiratory rate (bpm) | 18.8 (3.5) | 19.0 (3.6) | 0.276 | 0.05 |
| Temperature (℃) | 37.0 (0.5) | 36.9 (0.5) | <0.001 | 0.233 |
| Laboratory tests |  |  |  |  |
| WBC (×10^9^/L) | 12.1 (7.6) | 12.5 (9.0) | 0.174 | 0.042 |
| Hemoglobin (×10^12^/L) | 11.4 (2.0) | 11.0 (2.1) | <0.001 | 0.207 |
| Platelets (×10^9^/L) | 209.5 (93.9) | 203.5 (95.9) | 0.164 | 0.063 |
| Sodium (mmol/L) | 138.1 (3.7) | 138.4 (4.6) | 0.100 | 0.062 |
| Potassium (mmol/L) | 4.1 (0.6) | 4.3 (0.6) | <0.001 | 0.213 |
| Creatinine (mg/dL) | 1.1 (1.4) | 1.3 (1.4) | 0.319 | 0.039 |
| BUN (mg/dL) | 18.8 (15.4) | 23.6 (19.1) | 0.002 | 0.125 |

*SICU, Surgical Intensive Care Unit; CVICU, Cardiac Vascular Intensive Care Unit; TSICU, Trauma Surgical Intensive Care Unit; MICU, Medical Intensive Care Unit; MICU/SICU, Medical/Surgical Intensive Care Unit; CCU, Coronary Care Unit; NICU, Neuro Surgical Intensive Care Unit; SOFA, Sequential Organ Failure Assessment; SAPS II, Simplified Acute Physiology Score II; MAP, mean arterial pressure; WBC, white blood cell; BUN, Blood Urea Nitrogen.

**Table S4. Storage space consumption for each optimal model**

| Models | Storage space |
| --- | --- |
| Random Forest | 2.17MB |
| GBDT | 848kB |
| Adaboost | 75.9kB |
| Extra Trees | 1.4MB |
| Bagging | 2.26MB |
| MLP | 151kB |

*GBDT, Gradient Boosting Decision Tree; MLP, Multilayer Perceptron.

**Supplementary figures**


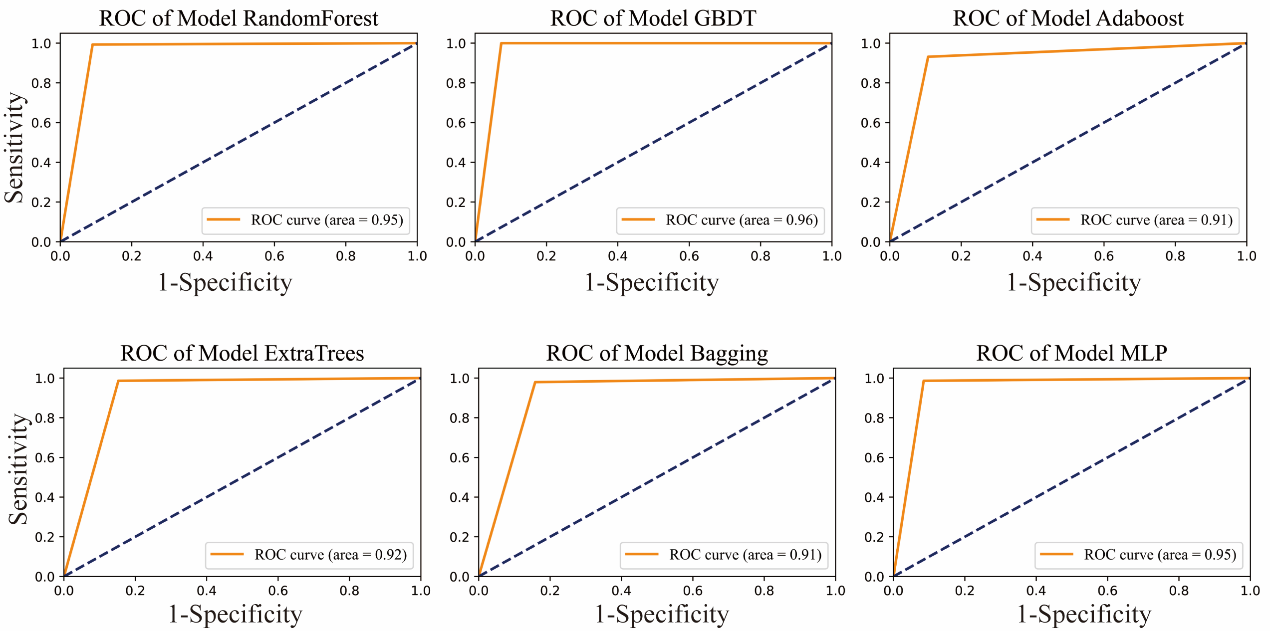


**FigS1. ROC curves of the testing cohort.**

**
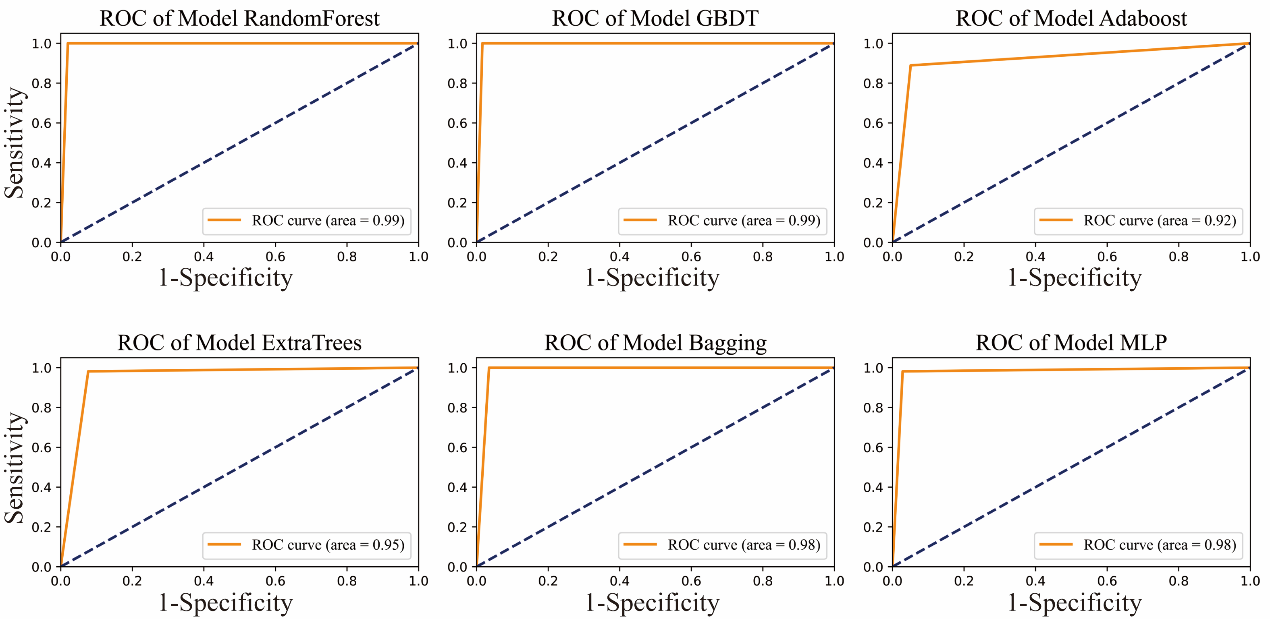
**

**FigS2. ROC curves of the validation cohort.**
